# Unified resilience model using deep learning for assessing power system performance

**DOI:** 10.1016/j.heliyon.2025.e42802

**Published:** 2025-02-19

**Authors:** Volodymyr Artemchuk, Iurii Garbuz, Jamil Abedalrahim Jamil Alsayaydeh, Vadym Shkarupylo, Andrii Oliinyk, Mohd Faizal Bin Yusof, Safarudin Gazali Herawan

**Affiliations:** a Department of Mathematical and Econometric Modelling, G.E. Pukhov Institute for Modelling in Energy Engineering of the NAS of Ukraine, Kyiv, Ukraine; b Department of Environmental Protection Technologies and Radiation Safety, Center for Information-analytical and Technical Support of Nuclear Power Facilities Monitoring of the NAS of Ukraine, Kyiv, Ukraine; c Department of Іnformation Systems in Economics, Kyiv National Economic University Named After Vadym Hetman, Kyiv, Ukraine; d Department of Computerized Control System, National Aviation University, Kyiv, Ukraine; e Department of Engineering Technology, Fakulti Teknologi Dan Kejuruteraan Elektronik Dan Komputer (FTKEK), Universiti Teknikal Malaysia Melaka (UTeM), 76100, Melaka, Malaysia; f Department of Computer Systems, Networks and Cybersecurity of the National University of Life and Environmental Sciences of Ukraine, Kyiv, Ukraine; g Department of Software Tools, Faculty of Computer Science and Technology, Zaporizhzhia Polytechnic National University, Zaporizhzhia, Ukraine; h Research Section, Faculty of Resilience, Rabdan Academy, Abu Dhabi, United Arab Emirates; i Industrial Engineering Department, Faculty of Engineering, Bina Nusantara University, Jakarta, 11480, Indonesia

**Keywords:** Deep learning, Energy resilience, Fidelity, Weather impact

## Abstract

Energy resilience in renewable energy sources dissemination components such as batteries and inverters is crucial for achieving high operational fidelity. Resilience factors play a vital role in determining the performance of power systems, regardless of their operating environment and interruptions. This article introduces a Unified Resilience Model (URM) using Deep Learning (DL) to enhance power system performance. The proposed model analyzes environmental factors impacting the resilience of batteries and energy storage devices. This deep learning approach trains performance-impacting factors using previously known low resilience drain data. The learning output is utilized to augment various strengthening factors, thereby improving resilience. Drain mitigation and performance improvements are combined for direct impact verification. This process validates the model's fidelity in enhancing power system performance, with a specific focus on the impact of weather factors.

## 1 Introduction

Power system resilience, which enables it to sustain an adequate energy supply, is one of the critical facilitators. Distributed Energy Resources (DERs) and smart grid technologies provide adaptability and real-time fault detection through integration [1]. Thus, modernization initiatives such as the grid will improve system efficiency and infrastructure. Demand response programs offer load-balancing support to help avoid failures during peak demand [2]. It is accomplished through energy storage systems that provide important backup power and grid stabilization [3]. In this, cybersecurity measures must be in place to safeguard critical infrastructure against ever-evolving threats. Redundancy and backup systems ensure continuity of operation during disruptions and further enhance resilience [4]. Regular personnel training and preparedness drills are critical to effectively responding to emergencies. The use of incentives to support investments in resilient infrastructure, given by regulatory bodies, brings necessary financing [5].

External factors, natural disasters, and extreme weather events largely affect the performance and resilience of the power system. Such events destroy physical facilities that contribute to interruptions [6]. Cybersecurity threats pose a considerably big threat, therefore necessitating solid protective measures. Regulatory frameworks control planning and development, affecting overall adaptability [7]. Global economic conditions and political events impact the availability of resources and energy costs [8]. Technological advancements offer opportunities but challenges in seamlessly integrating diverse technologies [9]. Environmental factors, such as climate change and air quality regulations, bring a shift towards the use of renewable energy sources. Population growth and urbanization affect electricity demand and infrastructure needs [10]. Public awareness and perceptions influence energy consumption patterns and efforts for sustainability. For better strategies in improving resilience, international collaborations and global energy trends contribute to shared knowledge [11].

Machine learning (ML) has been posed as a potent tool in enhancing power system resilience. Advanced algorithms in ML models unravel huge amounts of data in identifying patterns and predicting possible disruptions [12]. It facilitates real-time monitoring and detection of faults, allowing quick responses to issues and minimizing downtime [13]. Predictive analytics in ML can predict failures of equipment or cyber threats so that proactive mitigating strategies are made [14]. Adaptive learning in ML models allows the system to continuously learn and enhance its response to dynamic conditions. ML can optimize energy consumption patterns, supporting demand-side management and load balancing [15]. Improving grid control systems' accuracy in forecasting the electricity demand and supply through ML is an added feature [16]. In addition, anomaly detection by ML can quickly identify distortions in the system behaviour and thus detect the early occurrence of problems. ML offers a revolutionary approach that fortifies power systems against disruptions, making them more adaptive, efficient, and resilient [13,17]. The main contribution of the paper is.

⁃ Unified resilience model to estimate and provide recommendations for power system resilience improvement based on climatic and capacity impacts
⁃ Inheriting deep learning paradigm to validate and verify resilience based on power drain and identifying the strengthening factor for unification
⁃ Data-based representations, analysis, and validation using the proposed method
⁃ Comparing the proposed method through metrics with different methods and variants for competence detection.

The remainder of the study is prearranged as follows: section 2 deliberates the related works, section 3 discusses the data representation, section 4 discusses the proposed method, section 5 defines the Resilience and Performance Measurement, section 6 implements the performance assessment, and Section 7 shows the conclusion and future works.

## 2 Related works

Ali et al. [18] proposed an effective algorithm for the resilience of electric vehicle (EV) owners and multi-microgrids. The algorithm's main aim is to enhance EV owners' profit margin. A central energy management system (CEMS) gathers the necessary energy, which minimizes the latency in performing tasks. The important assets are utilized, and the grids increase the capability of the storage system. The proposed algorithm improves the performance and viability range of the systems.

Mokhtarzadeh et al. [19] developed an optimal planning model for resilience in smart residential microgrids (SRMG). It uses a two-state linearized mathematical programming framework to analyze the grid notions. The exact interactions between in-home energy management (i-HEM) are validated, producing relevant data for improvement. The developed model minimizes the SRMG's energy and computational costs.

Qin et al. [20] designed a hierarchical system of systems (SoS) using distributed energy scheduling for multi-microgrids (MMG). They analyzed the complex interaction behaviours to provide feasible resource scheduling services to the systems. The robust optimization model reduces the MMG's load-shedding range and enhances its overall resilience.

Zhao et al. [21] introduced a new resilience improvement approach for the distribution system (DS). Resilience improvement is a complicated task requiring proper resource allocation and scheduling processes. Operational and topological constraints are evaluated, producing optimal datasets for improvement. Experimental results show that the introduced approach improves the DS's resilience range.

Hosseinzadeh et al. [22] proposed a novel method for resilience improvement based on hybrid network zoning in DS. The actual goal of the method is to improve the capacity range of DS for the users. Limits and technical constraints are established for zoning, reducing each region's complexity. The constraints provide feasible information for resilience enhancement. The proposed method increases the effectiveness and feasibility level of the DS. Hou et al. [23] designed an integrated power distribution system (PDS) framework. The designed framework is mainly used to assess the extreme wind hazards presented in PDS. The component failures due to wind hazards are identified and eliminated by considering tree failures. It minimizes the computational cost level in the improvement process. The designed framework improves the overall resilience ratio of PDS.

Li et al. [24] introduced an unsupervised machine learning (ML) resilience method for power systems. The main aim of the method is to identify the fundamental properties of the power system. The identified properties are used as resilience curves, decreasing energy consumption in allocation and planning processes. The introduced method improves the feasibility range in the resource allocation prediction process. Saravi et al. [25] proposed a resilience-oriented planning framework for integrated distribution systems (IDS) and multi-carrier energy microgrids (MCEM). It is a cooperative planning framework that reduces the latency and energy consumption throughout the systems. It is used to consider the energy trading between the IDS and MCEM, which minimizes the cost of the systems. Compared with other frameworks, the proposed framework maximizes the energy exchange profit of the systems. Table 1 summarizes the rest of the references.

**Table 1:** Reference summary.

Author	Work	Features	Technique used	Results	Limitations
Al Kuwaiti et al. [26]	An optimized self-healing approach for power systems.	The actual aim is to enhance the resilience level of the networks.	Conservative voltage reduction (CVR) is used to reduce unnecessary demands.	Reduces the power consumption range while performing tasks.	Limited applicability in high-demand scenarios and dependency on accurate demand forecasting.
Chen et al. [27]	A two-layer optimal configuration approach for resilience enhancement in active distribution networks (ADN).	It is an energy storage system (ESS) that minimizes power fluctuations.	The k-means clustering technique is used here to analyze the network's fault scenarios.	Enhances the overall resilience range of the networks.	Complexity in analyzing large-scale fault scenarios and high dependency on accurate clustering outputs.
Lian et al. [28]	A resilience assessment index (REAI) for power systems.	It is mainly used to identify the damage and failures which are presented in the network.	A cascading failure graph is implemented here to evaluate and assess the curves in the networks.	Improves the performance and resilience range of the systems.	Requires extensive data on cascading failures, limiting real-time assessment capabilities.
Haghshenas et al. [29]	A cost-based optimization model power distribution network (PDN).	The main goal is to improve the resilience level under dust storms.	The exact possibilities of damages are identified, which minimizes the computational cost range of the networks.	Enhances the resilience level of the networks.	Limited to specific environmental conditions like dust storms, reducing generalizability.
Liao et al. [30]	A resilience evaluation method for the power system.	The proposed method is mainly used to enhance the capacity range of the systems.	The power source ability and network topology are considered for the power systems.	Maximizes the accuracy and resilience of the systems.	High dependency on accurate network topology data; limited scalability for larger networks.
Xu et al. [31]	A coordinated planning strategy for energy transportation network (ETN).	It is used for resilience enhancement, which minimizes the complexity range of the processes.	The primary energy sources are analyzed and provided to perform server tasks.	Improves the overall resilience range of the systems.	High computational demands during planning and limited applicability to diverse energy sources.
Wang et al. [32]	A machine learning-based technique (MLBT) for energy systems.	The main goal of the technique is to solve the power system problems presented in the system.	It is a quantification method that provides effective planning services to the systems.	Improves the sustainability and resilience of the energy systems.	It requires large datasets for training and may face challenges in dynamic and unforeseen conditions.
Sun et al. [33]	A new resilience assessment method for the integrated energy system.	The important features and patterns are evaluated to reduce the failure rate in the enhancement process.	Both spatial and temporal features are analyzed for the assessment process.	Enhances the resilience and feasibility ratio of the systems.	High data requirements for spatial-temporal analysis make implementation costly and time-consuming.
Huang et al. [34]	A resilience-oriented planning model for multi-energy system (MES).	It is a concurrent planning model that provides proper configuration for energy distribution.	Energy shifting is provided, which improves the resilience level of the systems.	Reduces the latency and energy consumption level of the systems.	Implementation challenges due to coordination complexities in multi-energy systems.
Venkataraman et al. [35]	Optimization algorithm for maintaining bufferless routing in low-power chip design	ALO-based buffer routing provides an operational frequency with power consumption.	The power dissipation of ant-lion optimized (ALO) routing topology consumes much less power.	Achieves an optimal operational frequency and minimized power consumption.	Limited scalability for more extensive and complex chip designs, restricting general applicability.
Kuthadi et al. [36]	Higher power efficiency techniques operate with sustained throughput and reliability levels.	Low energy pattern preservation and low power cyber safety modelling in smart grid applications	Integrating portable and data security tolerance-based energy-efficient frameworks identifies anti-nodes and key allocation with less energy.	Lower energy usage and improved throughput.	Dependency on energy-efficient frameworks may not address unforeseen energy distribution challenges in complex grid networks.

Reza Abshirini et al. [39] suggested a robust optimization approach for resiliency improvement in power distribution systems. The strategy incorporates demand response systems, mobile generators (MGs), and personnel teams for switching activities during restoration. The system's resilience is improved by using these aspects concurrently and considering the unpredictability of electricity demand and pricing. There are three levels to the goal function: minimum, maximum, and trilevel. First, the method considers the reconfiguration structure in power distribution networks and the position of MGs to reduce the commitment cost of combined heat and power plants (CHPs). Finding the most dire possible outcome for the uncertainty factors is the goal of the second level. The third and final level employs demand response techniques to reduce overall operating costs in the worst-case scenario. An IEEE 33-bus test distribution system is used to implement the suggested technique, and four examples are examined.

Mehrdad Mallaki et al. [40] recommended the multi-objective optimization approach for smart grid resiliency improvement. Due to the inaccuracy of classical models in predicting future power consumption, a new model is presented that utilizes quantile regression and twin support vector machines to make such predictions. A multi-objective optimization problem is laid out to reduce operating and investment costs while making the power grid more resilient. Numerical simulations are used to examine the performance of the suggested technique. The results show that resilience and dependability are improved when PV panels and batteries are sized and placed optimally. It has also been noted that overall investment and operating costs may increase because of global warming, and the power grid's resilience can be diminished.

Unlike traditional approaches, such as the conservative voltage reduction (CVR) technique, which primarily focuses on reducing power consumption yet struggles in high-demand scenarios, the URM provides a robust, data-driven framework capable of adapting to dynamic operational environments. Similarly, while the k-means clustering method effectively handles fault scenarios in active distribution networks (ADN), it lacks scalability for large-scale systems, a limitation addressed by the URM through its deep learning-based predictive capabilities. Compared to a cascading failure graph, which requires extensive failure data and is time-intensive, the URM offers real-time resilience assessment with reduced computational overhead. Furthermore, models like coordinated planning strategy, though effective in minimizing process complexity, often suffer from high computational costs, whereas the URM ensures computational efficiency through optimized deep learning architectures. However, the URM has challenges, including reliance on high-quality training data and potential difficulties integrating with legacy systems. The URM demonstrates superior adaptability, accuracy, and efficiency, significantly advancing resilience modelling for power systems.

## 3 Data representation

This article incorporates the popular “Western interconnection” (https://wimnet.ee.columbia.edu/portfolio/synthetic-power-grids-data-sets/) power grid network for statistical analysis. The network contains 14K + substations and 18K + distribution lines. The edge (power) distribution systems 26 are monitored to extract distribution and resilience impact. The resilience-impacting factors are climate and capacity. The region (with gridlines) and the distribution + resilience factors are illustrated below.

**Fig. 1:**
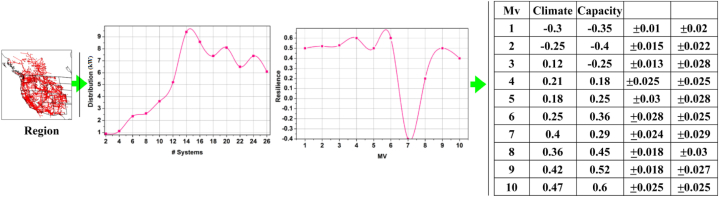
Region, distribution + resilience representation.

**Fig. 2:**
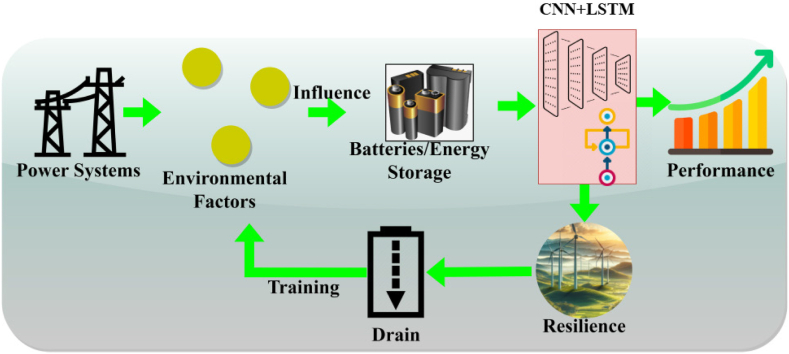
Representation of URM based on DL.

In the above Fig. 1, a region-based illustration is presented. The data shows the resilience with variance based on battery capacity and climatic impact. The performance is computed based on the varying resilience during the distribution hours. From the given data, the influence of climate factors over the distribution performance and energy drain is validated. The resilience index in Fig. 1 quantifies the system's ability to withstand and recover from disruptions caused by climate variability and energy demand. It is measured across different monitored values (Mv), with resilience values ranging from −0.3 to 0.6 (see Fig. 2).

The Western Interconnection power grid network's geographical scope is shown on the figure's left side, referred to as the region. It creates a map of the monitored distribution networks' gridlines and substations. The image below shows the region where the distribution and resilience analyses are used in perspective. The distribution performance (in kilowatts, kW) across 26 monitored power systems is shown in the figure in the center-left. To better understand the network's energy flow efficiency, it records how power is distributed and used across all of these components. The resilience meter measures how well a system can recover from disturbances (weather changes or energy consumption). It changes as a function of monitored values (Mv), representing the system's ability to work in various environments. The data in the table on the right shows the relationship between the monitored values (Mv) and variables like capacity (the ability to store energy or handle grid performance) and climate (the state of the environment that affects grid performance). The distribution performance and resilience are affected by the ±values, representing the margin of shifts in these aspects.

## 4 Unified resilience model (URM) using deep learning (DL)

Battery strengthening and energy resilience are vital in these broad economic and social impacting factors. The scope of this paper is to improve the performance of the battery and energy storage, which takes place in the context of environmental factors, including weather conditions. Here, the power system is built with the impact factor, which states the external factors such as climatic issues. Stability and sustainability are ensured in the batter, providing better performance than durability. Based on the impacting factors, energy resilience is used to assess natural disasters and provide efficient resilience. The countermeasure is taken regarding strengthening energy resilience in the power system. From this processing, the impacting factor reflects the battery drain and failure in the environmental factor. Hence, the control system is used to advance energy-strengthening energy resilience. A formal representation of the proposed URM based on DL is given below.

Energy resilience in renewable energy sources' dissemination components, such as batteries and inverters, is appreciated for achieving high operation fidelity. The resilience factors are optimal in deciding the power system's performance regardless of the operating environment and interruptions. In this work, deep learning is introduced to assess the battery life performance and resilience that ensure the energy in the battery. This article introduces the influences of the power system, which considers environmental factors for battery/energy storage. In this manner, the data analytics are integrated with the disaster in which the energy drain and resilience are observed for the battery's lifespan. The model is based on a convolutional neural network (CNN) combined with recurrent layers such as long short-term memory (LSTM) networks to capture spatial and temporal dependencies in system behaviour. The training involves supervised learning, using a dataset comprising historical power system data, simulated fault scenarios, and resilience metrics as labels. The research intends to assess the resilience of a regional power grid, such as one serving a population of 1 million, by modelling several failure scenarios and examining recovery trends. Data will be gathered from grid sensors, SCADA systems, and historical records of power outages. The grid's topology, including substations, power plants, and interconnection points, will be delineated to comprehend spatial characteristics. Temporal characteristics, like power fluctuations, system failures, and recovery durations, will also be included. The Convolutional Neural Network (CNN) will extract spatial patterns from the topology of the power system grid and sensor data, including voltage dips, current variations, and fault sites. The CNN will identify local characteristics, such as susceptibility regions within the grid. The Long Short-Term Memory (LSTM) network will identify temporal relationships, such as the impact of previous failures on the recovery process or how the system's performance amid severe weather conditions may affect future stability. The LSTM will manage sequential inputs, including power load fluctuations over time, system recovery sequences, and patterns of failure recurrence. Table 2 shows the nomenclature.

**Table 2:** Nomenclature.

Variable	Description	Unit of Measure
$E_{0}$	Integration of data analytics	–
$p_{m}$	The power system in renewable energy sources	MW (megawatts)
$E_{f}$	environmental factor	–
$v_{u}$	Vulnerability	–
$u_{r}$	Failure occurrence of a system failure or outage	–
$P_{i}$	prediction of energy storage and consumption	MW (megawatts)
$B_{y}$	batter level	% (percentage)
$G^{\prime }$	energy storage	MWh (megawatt-hours)
C	Checking the status, performance, or health of the power system or components.	–
μ	Identification	–
θ	Analysis of system behavior	–
$r_{0}$	Performance	% (percentage)
$d_{n}$	data analytics	Dimensionless (ratio)
α	Control process of system operations, such as adjusting power output or rerouting energy during failures.	%
$i_{a}$	Resilience	Dimensionless (ratio)
$D_{0}$	Battery drain	%/h (percentage per hour)
$p_{m}$	Climatic and storage factors	%
$I_{n}$	Distribution hours	Hours
T	Conditioning layer	Depth, thickness in physical terms
$s_{g}$	Strengthening Factors for resilience and operational capacity	%
$u_{i}$	Unification of different system components (e.g., energy sources, control systems)	0–1
$z_{h}$	synchronization to the timing or coordination of events or actions	timing index
ρ	Validation	0–1
φ	Recommendations for Power System Resilience Improvement	Qualitative index (e.g., “good”, “fair”, “poor")

The initial stage of this processing step is the integration of data analytics, which is equated in equation (1) below.

(1) E0=(pm+EfurPi+vu)∗∑Ef[(Pi+vu)∗(ur∗pm)]+(∑Ef(ur−vu)(pm+Pi))∗{[∫pmEf(Pi+ur)]−vu}

The integration of data analytics is executed above, and it is represented as $E_{0}$, the power system is labelled as $p_{m}$, $E_{f}$ is the environmental factor that includes climatic weather conditions. The vulnerability and failure are symbolized as $v_{u}\;and\;u_{r}$, in which it is associated with the prediction of energy storage and consumption and is formulated as $P_{i}$. In this case, vulnerability and failure are addressed, ensuring better energy storage performance. The integration of data analytics is used to provide the power system and ensure the battery lifetime (i.e., durability). Here, the impacting factor is associated with strengthening the battery life and advancing energy resilience. In this computation process, the influence is addressed, and reliable data analytics are provided. Indeed, power system resilience concerns are triggered by weather events that are severe enough. Hurricanes, heat waves, thunderstorms, and ice storms are more common and can cause widespread and recurring damage to power infrastructure, including outages and physical damage to transmission lines and substations, in contrast to geophysical disruptions like earthquakes and tsunamis. For resilience modelling to succeed, addressing these weather-induced issues is essential. These challenges need resilient systems that adjust quickly to offset disturbances successfully. Since this is the case, a thorough evaluation of power systems operation must consider weather influences when using the Unified Resilience Model (URM).

Data analytics are associated with vulnerabilities and failure in the environment. Here, the integration is used to analyze the vast amount of data from the energy storage system. Energy storage differs for every battery based on its strength and resilience. The integration is used to provide the vulnerability and failure for the batter resilience, and it is represented as (∑Ef(ur−vu)(pm+Pi)). In this point of view, the prediction is used to assess the power system better and deliberate the better-impacting factor in the proposed work. Based on this data analytic integration model, the checking is processed in the real-time and control system and formulated in the below equation (2).

(2) C=(pm+μ)∗((vu+ur)Ef∏dn(E0+pm))∗(∑Ef(Pi+By)[(r0∗μ)∗(G′+θ)])+∏r0By(G′+μ)+∑dn[pm+(G′+By)]

The checking is performed in the real-time and control system, and the power system to ensure the batter level and energy storage are described as $B_{y}\;and\;G^{\prime }$. The checking is C, the identification is formulated as μ, and θ is the analysis, which includes the impacting factors in the environment. The performance is $r_{0}$, here $d_{n}$ is data analytics. Based on the environment, the battery resilience is calculated. From this state of the art, the energy level is maintained in this computation step by considering the environmental factor. Here, the influencer provides the recommendation by examining energy storage. In this case, the energy storage is built with the prediction process that invokes the battery life and improves the energy storage. Algorithm 1 presents the C procedure towards performance measurement.

**Table:** Algorithm 1 C Procedure

$Inputs:B_{y},G^{\prime }$	
$Proceduce\;C\{B_{y},G^{\prime })$	
$Step\;1:for\;i=1\;to\;n\;\forall n\in p_{m}count$	
$Step\;2:compute\;E_{o}for\;p_{m}\;under\;E_{f}\;\forall dissemination\;Time$	
$Step\;3:if\;E_{o}>\left(u_{r}-v_{u}\right)\;then$	
$step\;4:P_{i}=E_{o}\left(u_{r}-v_{u}\right)\forall \;dissemination\;Time$	
$Step\;5:C=\frac{\prod_{r_{o}}^{B_{y}}\left(G^{\prime }+\mu \right)}{p_{m}};E_{o}=1$	
$Step\;6:else\;if\;E_{o}\leq \left(u_{r}-v_{u}\right)then$	
$Step\;7:r_{o}=\frac{E_{o}}{E_{o}+p_{m}}\;\forall \;B_{y}=\max\;and\;G^{\prime }=B_{y}$	
$Step\;8:C=\left(\frac{P_{i}+B_{y}}{p_{m}}\right)\;\forall \;C>C\left(E_{o}-1\right)$	
Step9:Endif	
Step10:Endfor	

In the steps above, the checking is executed in a real-time environment where the control system extracts the necessary energy from the battery. The influences are associated with the impacting factor by deliberating this energy consumption function. This impacting factor derives from the vulnerabilities and failure of the battery energy and drains. This draining of the battery level and energy storage is associated with the prediction model. The prediction is used to examine the resilience of the battery and improve the standard level of performance. The performance is improved in this category for the power system, and it is formulated as $\sum_{d_{n}}\left[p_{m}+\left(G^{\prime }+B_{y}\right)\right]$. The environmental factor is realistic for the power system and is derived in equation (3) below.

(3) Ef=(α+μ)∗∏pm(ur−vu)+By∗G′+((By+G′)[(r0+pm)∗((ur−vu))])∗(∑dn(Pi+By)(G′∗θ))

The environmental factors are associated with the power system, which predicts the battery level; the control process is labelled as α. The principal issue here is the climatic condition of the energy storage battery. In this manner, the vulnerabilities and failures are addressed and reduced. The environmental factor provides the device's battery level, storage space, and resilience. Thus, the concept is to discuss the power system's validation by deploying the battery life performance. The battery level is maintained in this process and executes without an energy level drain. In this computation step, the power system is used to influence better the battery resilience in which it executes the data analytics.

The environmental metrics are considered for examining storage and battery life under the distribution recommendation system. Here, the vulnerabilities are associated with the prediction model that deploys the resilience of the battery storage. The storage of the battery level ensures the prediction of the energy in the battery. In this step, the vulnerabilities and failures are associated with the performance of the data analytics. This step includes checking the battery in real time and deploying the power control system. Here, it derives the prediction process for the energy storage and performance for the environmental factors and improves the power system, and it is represented as $\left(\frac{\left(B_{y}+G^{\prime }\right)}{\left[\left(r_{0}+p_{m}\right)*\left(\left(u_{r}-v_{u}\right)\right)\right]}\right)$. The following equation (4) derives the influence analysis from this computation step.

(4) In=(∑Pi(By+G′)ia−D0)∗((r0+By)d0C+Ef)+∏dn[(θ∗α)+((r0+G′)[(ia−D0)+By])]−(vu−ur)+α∗[(r0∗Pi)]

**Fig. 3:**
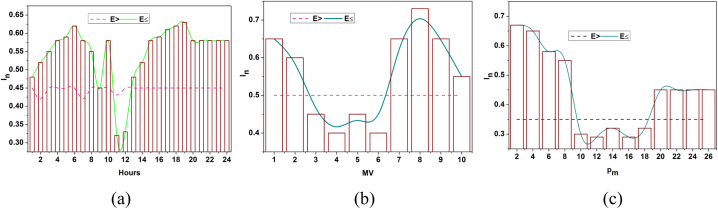
Influence analysis for Dissemination (a) Hours, (b) Capacity, and (c) Systems.

The influences are formulated above, and it is represented as $I_{n}$, in this computation step, the prediction of battery level and drain is associated with the energy storage in the previous step. Resilience is represented as $i_{a}$, here $D_{0}$ is described as battery drain. In this analysis, the performance level is built with advanced strengths in battery durability. The direct impact of climatic and storage factors over the $p_{m}$ is analyzed in Fig. 3, and this analysis is based on the average dissemination (a) hours, (b) capacity, and (c) systems experienced.

The $I_{n}$ for distribution hours, capacity, and $p_{m}$ are directly influenced by climatic and storage factors. The $I_{n}$ is different from the conventional observed values as in Fig. 1. The values differ from the provided and estimated values with definite variance. The $E<\left(u_{r}-v_{u}\right)$ and $E\leq \left(u_{r}-v_{u}\right)$ conditions are validated in the above representation. This difference shows both high and low variance that requires optimal μ∀θ. Therefore $i_{a}$ is dotted line observed for $B_{y}$ and $G^{\prime }$ over the $r_{o}$ (Refer to Fig. 3). Here, the identification is based on the data analytics in which the resilience is associated with battery drain. This evaluation step addresses the vulnerabilities and failures by examining this influence level. The data analytics is used to deploy the impacting factors proposed for the resilience, and the battery level is drained. Here, the recommendation validates the battery level and provides a reliable output. In this case, the performance is improved in which the resilience and the draining state are observed for the storage and the battery level, and it is represented as $\left[\left(\theta *\alpha \right)+\left(\frac{\left(r_{0}+G^{\prime }\right)}{\left[\left(i_{a}-D_{0}\right)+B_{y}\right]}\right)\right]$. Here, both the impacting factors are influenced in this environmental case, and based on this observation, the prediction for the resilience of the batteries and storage is examined. From this presentation model, the battery/energy storage is used to deploy the integration of the data analytics. In this manner, the recommendation is performed for the control system associated with the environmental factors. In this case, battery/energy storage identification is detected in the equation below (5).

(5) $$\mu =p_{c}+\left(v_{u}-u_{r}\right)*\sum_{P_{i}+B_{y}}\left(r_{0}*i_{a}\right)+\left(E_{0}*\frac{G^{\prime }+B_{y}}{I_{n}}\right)$$

The identification is used to improve performance in the proposed work. In this concept, the impacting factors are associated with resilience and battery drain. The battery drain is detected in this processing step, in which the prediction is managed. The prediction is followed up with the previous energy level and improved based on this performance level. The influence is associated with the environmental factor in which the identification is processed for resilience, and it is formulated as $\sum_{P_{i}+B_{y}}\left(r_{0}*i_{a}\right)$. Hence, the identification is directly associated with the prediction process in which data analytics is deployed for the power system. The $D_{0}$ outputs across dissemination hours, capacity, and systems are statistically presented below.

**Fig. 4:**
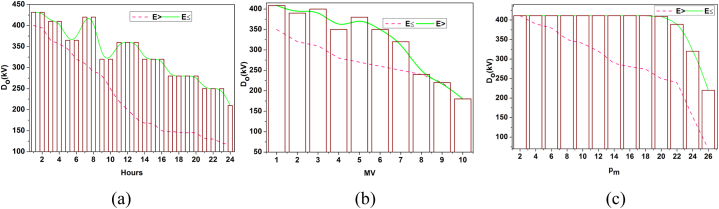
Battery drain for Dissemination (a) Hours, (b) Capacity, and (c) Systems.

Fig. 4 shows the battery drain for dissemination (a) hours, (b) capacity and (c) systems. The drain variant is impacted by $E>\left(u_{r}-v_{u}\right)$ and $E\leq \left(u_{r}-v_{u}\right)$ constraints experienced per $p_{m}$ per distribution time. The performance estimation is the cumulative consent using $I_{n}$ and $D_{o}$. Both factors are used to identify $i_{o}$ that is different from the criteria represented. However, the learning process defines the range of variations of resilience to prevent interrupted failures (Fig. 4). The periodic checking is examined in this step, which is associated with predicting and identifying the storage space. The battery standup level determines the energy level and the storage space. The durability is given for certain batteries and checked in a real-time environment. Here, the vulnerability and failure are directly interlinked with the impacting factor that addresses them and maintains the battery level. From this point of view, the power system is used to examine the advancements in evaluating battery strengths. Post to this identification method, the deep learning concept is introduced for energy storage/battery resilience and draining state detection.

## 5 Resilience and performance measurement

The interconnection among the input and output neurons in which the forwarding is carried out in the n-layers. This concept is built with the range of neurons that defines the multiple layers. The characteristic of this deep learning is the hierarchical feature, which improves the data computation. In this method, energy resilience and draining are examined to improve performance. The recommendation is enhanced by the environmental factors associated with energy draining and resilience in the power system. The following equations (6), (7) are used to find the resilience and draining state of the battery.

(6) $$i_{a}=P_{i}+\left(B_{y}+G^{\prime }\right)*\left(E_{f}+\theta \right)*\mu$$

Whereas,

(7) $$D_{0}=P_{i}-\left(B_{y}+G^{\prime }\right)*I_{n}+\alpha$$

Both equations (6), (7) are used to ensure the performance of the battery level, considering that the battery storage and level are associated with the impacting factors. In this manner, the prediction maps the current and previous processing and gives the appropriate result. Here, the association is termed for energy storage and battery-level maintenance. Hence, this concept is used to examine the recommendation based on the data analytics and the better recommendation improvement. This deep learning is used here with the distinct layers in which it forwards the resilience and drain-free data to the next layer. In this methodology, the recommendation is the output in which better performance is observed. The conditional layer is designed to envelop the training for further computation in the deep learning concept from these derivations in equation (8).

(8) $$\begin{array}{c} {B_{y}\left(0\right)=p}_{m}+\left(v_{u}-u_{r}\right)*\prod_{P_{i}}^{\theta }\left(\mu *\alpha \left(0\right)\right)\\ {B_{y}\left(1\right)=p}_{m}+\left(v_{u}-u_{r}\right)*\prod_{P_{i}}^{\theta }\left(\mu *\alpha \left(1\right)\right)\\ \vdots \\ {B_{y}\left(n\right)=p}_{m}+\left(v_{u}-u_{r}\right)*\prod_{P_{i}}^{\theta }\left(\mu *\alpha \left(n-1\right)\right) \end{array}\}$$

The conditional layer is used in this deep learning to train the certain battery status to improve resilience and avoid the draining state. In this manner, $B_{y}\left(0\right)$ is the first battery taken into processing, whereas, $B_{y}\left(n\right)$ of batteries are considered in this paper. Here, the control system acts for the battery usage where the initial system is defined as α(0), α(n−1) is the distinct control system used in this computation step, as shown in equation (9).

(9) $$T=\theta +p_{m}-\left(G^{\prime }+B_{y}\right)+C\left(\alpha \left(n-1\right)\right)$$

The training phase is introduced in this work where the performances state the impacting factor that deploys the conditioning layer for this execution, and it is described as T. Here, the control system indicates the distinct layers in the deep learning; if any vulnerability or failure occurs, then the drop in the battery level is stated. So, to avoid this, the training phase is used to train the failed metrics in the process and avoid the further processing step, which is forwarded to the interlinked neuron in the network. The learning procedure is described in Algorithm 2.

**Table:** Algorithm 2 Learning Procedure for $r_{0}$

$Input:procedure\;r_{o}Learning\;\left\{i_{a},G^{\prime }\right\}$	
$Step\;1:Initialize\;\left(c,\theta \;\forall p_{m}\right)$	
$Step\;2:while\;I_{n}\;!=0\;do\{$	
$Step\;3:Compute\;i_{a}=P_{i}+\left(B_{y}+G^{\prime }\right)*\left(E_{f}+\theta \;\right)+\mu$	
$Step\;4:if\;i_{a}<\left(I_{n}+\alpha \right)then$	
$Step\;5:B_{y}\left(n\right)=p_{m}+E_{o}*\left[\alpha \left(n-1\right)\right]$	
$Step\;6:Estimate\;r_{o}\;as\frac{I_{n}}{B_{y}\left(n\right)}\;such\;that\;E_{f}=I_{n}+i_{a}$	
$Step\;7:Else\;if\;i_{a}>\left(I_{n}+\alpha \;\right)then$	
$Step\;8:b_{y}\left(n\right)=I_{n}+\left(E_{o}-1\right)*\left(\frac{\alpha }{p_{m}}\right)$	
$Step\;9:Estimate\;r_{o}\;as\;I_{n}+\frac{i_{a}}{B_{g}\left(n-1\right)}\;such\;that\;E_{f}=i_{a}$	
Step10:break;	
$Step\;11:if\;i_{a}=\left(I_{n}+\alpha \right)then$	
Step12:RepeatfromStep4	
Step13:Endif	
Step14:Endwhile	

Based on this training, the resilience and the drain of the battery are observed, and the strengthening factor is considered by equating the following equation (10).

(10) $$s_{g}=\sum_{p_{c}}\left(r_{0}*\left(G^{\prime }+B_{y}\right)\right)+\left(\frac{P_{i}+\theta }{\mu }\right)*I_{n}$$

The strengthening is considered for the energy resilience in which it deploys the battery/energy storage. Here, the performance is observed in this category, which poses energy storage and battery usage. The strengthening factor is labelled as $s_{g}$, which states the environmental condition of battery sustainability. In this case, prediction is followed up to identify the influence of this power system. Based on this examination factor, the analysis is carried out for the different impacting metrics in this work. Post to this, synchronization is provided for the unification of drain mitigation and performance improvement, whereas validation is examined in equations (11), (12) below.

(11) $$z_{h}=\left(u_{i}+r_{0}\right)*\prod_{\theta }\left(\mu *s_{g}\right)+\left(P_{i}+T\right)$$

Whereas,

(12) $$\rho =\left[\left(T+\mu \right)+\left(i_{a}+D_{0}\right)\right]*\left(P_{i}+r_{0}\right)$$

**Fig. 5:**
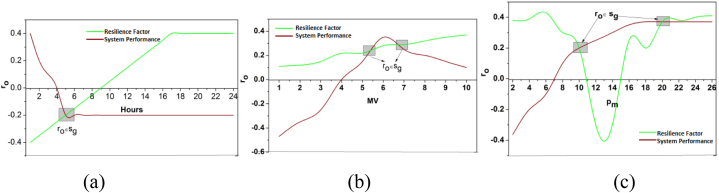
(a) Distribution over hours (b) Distribution vs MV (c) Distribution vs $P_{i}$

The synchronization and validation are derived in the above equations (11), (12), and they are represented as $z_{h}\;and\;\rho$. In this process, unification is $u_{i}$, in which it deploys the prediction for this proposed work. Here, it deploys the strengthening of the battery storage and avoids vulnerability and failure. After the training process, the converging point for the two influencing factors is identified in Fig. 5.

Fig. 5 shows the composite visualization showing (a) the variation of the resilience factor $r_{0}$ (green) and system performance $f_{g}$ (red) over hours, (b) the relationship between resilience factor $r_{0}$ and MV, and (c) the interaction of the resilience factor $r_{0}$ with $P_{i}$.

Fig. 5(a) Distribution over hours shows how the resilience factor and system performance vary over time. Fig. 5(b) Distribution vs MV depicts the relationship between MV and the resilience factor/system performance. Fig. 5(c) Distribution vs $P_{i}$ illustrates the variation of the resilience factor and system performance concerning $P_{i}$.

The $r_{o}\in s_{g}$ is the convergence of $D_{o}$ and $i_{a}$ for hours, MV, and $p_{m}$ variances. The $B_{y}\left(n\right)$ differentiation is performed for α(n−1) or $\left(\frac{\alpha }{p_{m}}\right)$ such that $\left(i_{a},G^{\prime }\right)$ is observed. If the observation is classified under step 4 or step 7 of algorithm 2, then the convergence is either incremented/decremented towards E≥orE< conditions. The convergence is verified for the illustrations in Fig. 1, for which $I_{n}$ and $i_{a}$ are analyzed for capacity and $D_{o}$. As convergence is achieved, performance and resilience are easily analyzed (Fig. 5). This integration provides data analytics for impacting factors. This computation is used to state the power system of the resilience and examine the synchronization for the unification in drain mitigation of the performance and sows for better improvement. Validation enhances the performance of examining the prediction identification for the training phase. This deep learning is associated with the training phase for the validation process. The following part provides the performance improvement recommendation in equation (13) below.

(13) $$\varphi =\left[\left(\rho +r_{0}\right)+\left(i_{a}+D_{0}\right)\right]*T\left(G^{\prime }+B_{y}\right)$$

**Fig. 6:**
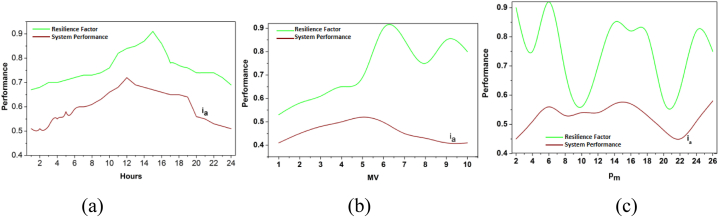
System performance analysis (a) Performance vs Hours (b) Performance vs MV**(c) Performance vs**${\;P}_{i}$

The recommendation is φ is provided for the performance improvement by executing the validation and synchronization. From this process, the impacting factors are associated with the influence analysis and avoid further steps in the neural network. This deep learning deliberates the training phase that strengthens the battery energy and improves durability. If there is any resilience or it drains the battery, then the training is executed in the neural network and improves the performance and recommendation in the environmental factor. In this process, the learning is induced to validate its fidelity towards power system performance. This proposed model is designed based on the impact of weather factors on power systems. Based on $z_{h}$, the performance and resilience are further validated in Fig. 6.

Fig. 6 shows the System performance analysis. (a) Variation of performance metrics $r_{0}$ (green) and $r_{0}$ (red) over hours; (b) Relationship between performance metrics and MV; (c) Interaction of performance metrics with parameter ${\;P}_{i}$. The assessment of performance and $i_{a}$ for different hours, MV(capacity), and $p_{m}$ are assessed in the above representation (Fig. 6).

Fig. 6 (a) Performance vs Hours analyzes system performance and resilience factors over time. Fig. 6(b) Performance vs MV explores system performance and resilience factors relative to MV. Fig. 6(c) Performance vs ${\;P}_{i}$ compares system performance and resilience factors concerning ${\;P}_{i}$.

The $B_{y}\left(n\right)$ or $B_{y}\left(n-1\right)$ differences for $r_{o}$ and $i_{a}$ detection generates $s_{g}$ for distribution. Such distribution is validated for $E\leq \left(u_{r}-v_{u}\right)$ (or) $E>\left(u_{r}-v_{u}\right)$ conditions to integrate multiple options for performance. Hence, in this case, the actual and computed validations changes are converged. If the $s_{g}$ is observed in any hours/MV or $p_{m}$ then it is benchmarked for resilience. Therefore, this remains until the final performance measure is computed. Fig. 6 shows the correlation between resilience (red line) and performance (green line) as a function of three variables: power distribution systems (*Pm*), monitored values (Mv), and hours of operation. Performance and resilience change over a day in the first graph; the former peaks at peak demand times, while the latter trails slightly behind, suggesting the system's capacity to recover from heavy loads. Performance and resilience vary among monitored values (Mv), as seen in the second graph. At performance peaks, resilience decreases, perhaps because the system is under more stress. The final graph compares these measures with 26 other distribution systems (*Pm*), revealing which systems perform well and have trouble staying resilient in the same environments.

## 6 Performance assessment

This article proposes a Unified Resilience Model (URM) using Deep Learning (DL) to improve power system performance. The suggested URM model analyzes environmental factors impacting the resilience of batteries and energy storage devices. This DL approach trains performance-impacting factors utilizing previously known low resilience drain data. The learning output is used to augment various strengthening factors, enhancing resilience. Apart from the data-oriented analysis, a comparative assessment is presented below to compare the performance of the proposed method with similar existing methods. In this regard, the existing CVR-MINLP [26], CROPF [25], and RO-OMS [20] methods from the related works are inherited for assessment. These methods conjointly analyze the performance factor, impact analysis, resilience factor, drain, and analysis time metrics. The metric's influence by increasing the capacity (MV) and systems ($p_{m})$ is studied.

**Fig. 7:**
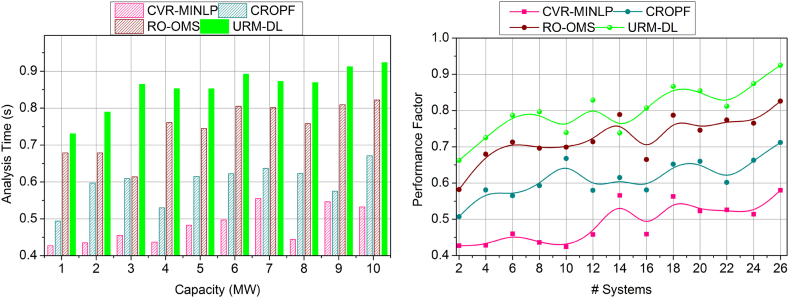
Performance factor assessment by (a) increasing the capacity and (b) systems.

Fig. 7 shows the performance factor for the proposed work increases for battery capacity and the number of systems. Here, the discussed metric illustrates the energy resilience of the battery without any draining state. In this state, real-time checking is followed up for the battery energy resilience in which it acquires environmental factors such as weather conditions. Here, the impacting factors are vulnerabilities and failure of the control system in which the battery drain is detected. The strengthening of the battery is used to define better performance among the environmental factors. Here, the power system acquires the energy for the particular battery, which it processes in the required period, and then draining is defined. In this stage, the performance factor is addressed and improves the durability of the battery lifetime and is illustrated in the validation step, and it is formulated as $\left[\left(T+\mu \right)+\left(i_{a}+D_{0}\right)\right]$. In this manner, the environmental factors are taken into consideration, and based on this, drain mitigation is examined in this performance factor, which shows better improvement in this proposed work.

**Fig. 8:**
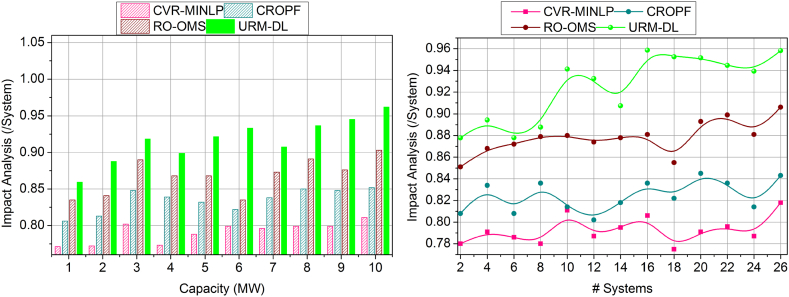
Impact analysis assessment by (a) increasing the capacity and (b) systems.

The impact analysis is higher in Fig. 8, which is processed based on the metrics of battery capacity and number of systems. The proposed work concentrates on the impact analysis and shows a higher range in which it falls on external factors such as climatic conditions. In this phase, deep learning is introduced in this concept to fix the impact analysis in this proposed work. Based on this processing, data analytics is integrated to define battery/energy storage in a real-time system. In this manner, resilience analyses the battery status and provides reliable computing during draining. In this manner, the validation process provides recommendations that address the vulnerabilities and failures of this work. The formulation is computed on the environmental factors to detect the impact analysis, and it is equated as $\left(v_{u}-u_{r}\right)+\alpha *\left[\left(r_{0}*P_{i}\right)\right]$. Thus, the impact analysis is integrated with the data analytics and shows better performance among the control systems where the battery capacity is the benchmark for the proposed work.

**Fig. 9:**
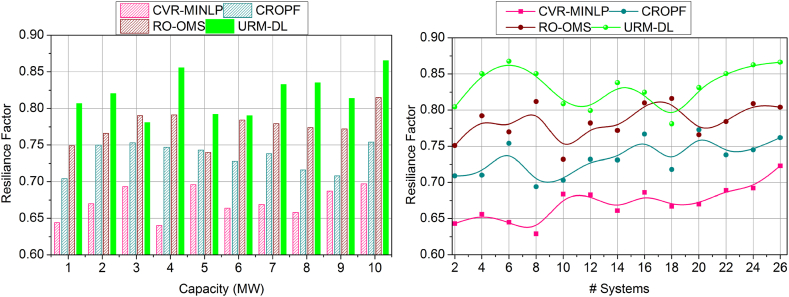
Resilience factor assessment by (a) increasing the capacity and (b) systems.

The resilience factor is enhanced in this proposed work, shown in Fig. 9, where the battery capacity and number of systems are considered. Here, the resilience factor is considered for the battery durability with lesser drain occurring on environmental factors. This analysis is carried out to identify battery/energy storage that occurs in real-time. In this deployment, the power system is developed to store the energy resilience where the battery durability is extended. Here, the training phase is introduced in this deep learning, where the influences are detected and avoided in the further neural network. Here, the interlinked layers are associated with the conditional layer processing in which the training is processed, and it is represented as $\left(G^{\prime }+B_{y}\right)+C\left(\alpha \left(n-1\right)\right)$. The distinct layers are associated with the control system, which deliberates the impacting factor. The training phase trains the impact factor to improve the resilience of the battery in a better manner. In this stage, checking is processed for the real-time system, which deploys the strengthening of the battery life span and shows a better resilience factor.

**Fig. 10:**
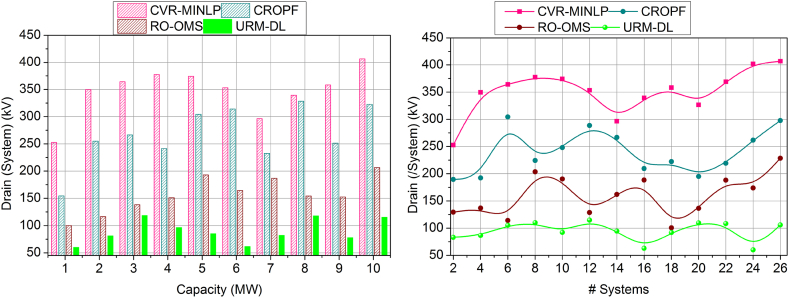
Drain assessment by (a) increasing the capacity and (b) systems.

The battery drain is illustrated in Fig. 10, which shows less of this proposed work that plots on variants such as the number of systems and capacity of batter level. Based on this processing, energy storage is developed, and performance is also improved. This computation uses resilience and energy storage to identify the synchronization of the unification in drain mitigation and performance improvement. In this part, the drain is reduced by detecting the impact factors in which the training is used to provide performance-level influences. Here, the battery drain is used to define the power system. Here, the analysis is followed up with the system's recommendation for the battery performance and delivery of the drain-less battery. This work improves performance for the varying resilience battery, showing a better-impacting factor. Here, the analysis is carried out for energy storage and improves the strengthening of the battery, and it is formulated as $\left(B_{y}+G^{\prime }\right)*\left(E_{f}+\theta \right)*\mu$. In this processing step, validation is provided to address the battery drain, which provides less in this work.

**Fig. 11:**
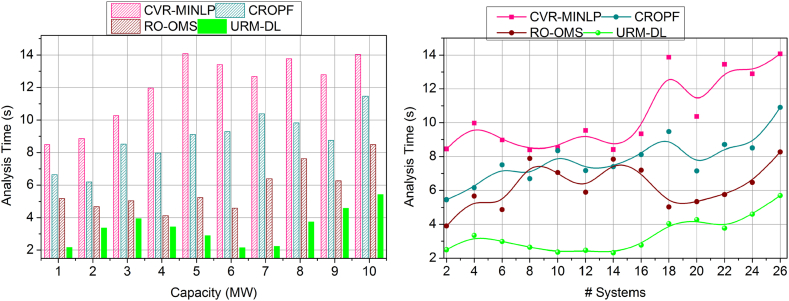
Analysis Time assessment by (a) increasing the capacity and (b) systems.

Fig. 11 shows the lesser analysis time in this proposed work for the capacities and varying number of systems. Here, it deploys the impacting factor that illustrates the resilience of the battery, and based on this, the strengthening is processed to avoid the vulnerabilities and failure of the battery. The analysis time is calculated for the performance and recommendation of the battery, which improves the resilience. Based on this, draining is reduced. The synchronization is processed based on the prediction where the influences are addressed in these impacting factors. Here, the training is performed for the strengthening factor, and the checking is observed for the control system in real-time. In this case, the identification is carried out for the analysis time to find the resilience and battery drain, and it is equated as $\left[\left(\rho +r_{0}\right)+\left(i_{a}+D_{0}\right)\right]$. The validation is carried out for the resilience of the battery and drains state and, from this time, is calculated for better performance and recommendation. Thus, the analysis time is estimated to be the lesser duration for the battery resilience in this proposed work. The above discussion is summarized based on the improvements achieved by the proposed model.

o ***Variable:*** Capacity|| Performance Factor (12.42 % Improved), impact analysis (10.66 % Improved), resilience factor (10.99 % Improved), Drain (10.51 % Reduced), and analysis time (8.7 % Reduced).
o ***Variable:*** Systems|| Performance Factor (10.9 % Improved), impact analysis (10.25 % Improved), resilience factor (10.33 % Improved), Drain (11 % Reduced), and analysis time (8.11 % Reduced).

Although information may not always be consistently accessible across real-world systems, the model relies on high-fidelity input data, such as precise battery performance indicators and network specifications. It may want modification for intricate or non-standard power networks as it is optimized for conventional topology. The model considers charge-discharge dynamics but fails to completely account for non-linear behaviours under severe circumstances or long-term deterioration. At this level, environmental elements like weather and natural catastrophes are considered, but the degree of detail needed may be lacking in cases where they have a dominant role. Furthermore, the model's performance indices depend on assumptions of steady network connectivity, which may not be applicable in all cases, and the computing difficulties that may arise from scaling the model to very large systems are another concern. In light of these factors, it is clear that further work and application-specific adjustments are needed to be more resilient and applicable in the real world.

Implementing the proposed Unified Resilience Model (URM) in real-world scenarios faces several challenges, as the first step in integrating the model with the current power grid infrastructure is to enhance the data-gathering systems. This includes installing real-time sensors and advanced monitoring tools to gather the data needed for climatic and capacity analysis. Furthermore, not all locations may have access to the computing resources and strong data management systems needed to train the model using large-scale datasets for deep learning. Finally, guaranteeing the model's flexibility and accuracy under varied situations is made more difficult by the variety and unpredictability of severe weather occurrences. Finally, it is critical and hard to achieve the involvement of stakeholders, which includes utilities, legislators, and regulatory organizations. This is because it involves coordinating technical, financial, and policy objectives.

Traditional resilience models based on statistical time series forecasting (e.g., ARIMA or SARIMA) will be compared to the URM model. The CNN-LSTM model and the conventional approaches will have their mean prediction errors compared using a paired *t*-test. The model's accuracy in forecasting system failures will be evaluated by calculating its sensitivity, which is the true positive rate, and specificity, which is the true negative rate. This will ensure that the model doesn't generate excessive false positives. Confidence intervals will be calculated around the expected recovery timeframes to determine how uncertain the model's predictions are. The model's performance may be tested by running Monte Carlo simulations with different input parameters, such as weather data or the reliability of the sensors.

## 7 Conclusion and future work

This article introduces and discusses the unified resilience model (URM) using deep learning (DL), such as CNN with LSTM approaches. The proposed model was designed to analyze, validate, and recommend resilience-based improvements for power systems. The proposed model was validated using an external dataset incorporating deep learning to identify the impacts of the environment and capacity. This learning process is used to identify low resilience impacted by climatic and capacity influences both jointly and independently. The network is trained to identify augmenting factors based on the learning identification for strengthening factors towards resilience improvement. Depending on these external impacting factors, the power system's performance is computed to identify its resilience between continuous distribution hours. This model was compatible with the direct, indirect, and unified influencing factor assessment. Under the variable capacity, Performance Factor (12.42 % Improved), impact analysis (10.66 % Improved), resilience factor (10.99 % Improved), Drain (10.51 % Reduced), and analysis time (8.7 % Reduced) are the comparative assessment results. Data points like recovery times and load demand show that the system can swiftly settle down after disruptions, proving that the model can optimize resilience. By demonstrating a few cascade failures, fault tolerance statistics prove that the model successfully identifies and reduces the impact of critical situations. Voltage and frequency stability measures also show fewer variations when interruptions, which shows that the URM can keep operations running smoothly.

The proposed model is reliable in assessing and rectifying the resilience-impacting issues in power storage and distribution systems. Though it handled the power storage systems' joint influence factor, the peak distribution resilience problem is less considered in this proposed model. Therefore, the proposed model will optimize preventive distribution using multi-objective decisions. This direction is particularly relevant in the context of new threats to energy systems, as highlighted by recent works on flexible grids with small modular reactors [37] and structurally variable electric power systems resistant to terrorist and military threats [38].

## CRediT authorship contribution statement

**Volodymyr Artemchuk:** Software, Investigation, Conceptualization. **Iurii Garbuz:** Validation, Project administration, Investigation. **Jamil Abedalrahim Jamil Alsayaydeh:** Writing – original draft, Validation, Resources. **Vadym Shkarupylo:** Formal analysis, Conceptualization. **Andrii Oliinyk:** Writing – review & editing, Methodology. **Mohd Faizal Bin Yusof:** Writing – review & editing, Funding acquisition. **Safarudin Gazali Herawan:** Writing – original draft, Funding acquisition.

## Data availability statement

The popular “Western interconnection” (https://wimnet.ee.columbia.edu/portfolio/synthetic-power-grids-data-sets/) power grid network is incorporated in this article for statistical analysis. Other data for this article will available on Zenodo:

https://zenodo.org/records/14854355.

## Declaration of generative AI and AI-assisted technologies in the writing process

During the preparation of this work, the author(s) used OpenAI's language model, ChatGPT, to verify the text and check for errors. After using this tool, the author(s) reviewed and edited the content as needed and take(s) full responsibility for the content of the publication.

## Declaration of competing interest

The authors declare that they have no known competing financial interests or personal relationships that could have appeared to influence the work reported in this paper.
